# Psychosocial factors and caregiver burden among primary family caregivers of frail older adults with multimorbidity

**DOI:** 10.1186/s12875-023-01985-y

**Published:** 2023-01-30

**Authors:** Cheuk Ying Chan, Jacqueline Giovanna De Roza, Gabriel Teck Yong Ding, Hui Li Koh, Eng Sing Lee

**Affiliations:** 1 National Healthcare Group Polyclinics, Singapore, Singapore; 2 Lee Kong Chian School of Medicine, Nanyang Technological University, Singapore, Singapore

**Keywords:** Psychosocial, Caregiver burden, Frail, Multimorbidity

## Abstract

**Background:**

Provision of care for frail older adults with multiple chronic diseases (multimorbidity) poses increasing challenge for family caregivers. Our study aims to evaluate to what extent caregiving competence, social support and positive aspects of caregiving can mitigate the effect of burden experienced by family caregivers of frail older adults with multimorbidity.

**Methods:**

A descriptive cross-sectional study was conducted in 2 primary care clinics. Family caregivers of older adults aged 65 years and above were invited to complete interviewer-administered questionnaires. Descriptive statistics were used to describe sociodemographic and clinical data. Caregiver’s burden was measured using the Zarit Burden Interview (ZBI). Mann–Whitney U test was used to compare differences in Caregiving Competence Scale (CCS), short Positive Aspects of Caregiving (S-PAC) and modified Medical Outcome Study Social support (mMOS-SS). Multivariable logistic regression was used to analyse factors associating with caregiver burden.

**Results:**

A total of 188 participants were recruited. 71.8% reported caregiver burden (ZBI score ≥ 10). Caregivers who perceived burden had significantly lower CCS, S-PAC and mMOS-SS scores than those who did not (10.0 vs 11.6; 26.8 vs 29.8; 24.8 vs 31.4, *p* < 0.001 respectively). Factors significantly associated with higher odds of perceived burden were presence of alternative caregivers (OR 3.3, 95% CI 1.09, 10.19, *p* = 0.04), use of community resources (OR 4.4, 95% CI 1.15, 16.83, *p* = 0.03) and time spent caregiving per week (OR 1.1, 95% CI 1.02, 1.10, *p* = 0.003).

**Discussion and conclusion:**

This study found that caregivers had high perception of burden as demand in caregiving may increase. Anticipating caregiver burden and social support needs may be important part of managing these frail older adults.

## Introduction

Provision of care for frail older adults with multiple chronic diseases (multimorbidity) poses increasing challenge for family caregivers. Frailty, in addition to multimorbidity, can result in increased family caregiver burden especially when increased time spent caregiving was necessitated [1]. Several studies have shown evidence of caregiver burden especially in caring for older adults with increasing dependency [2–4]. For older adults with frailty, the loss in body reserves as a result of exposure to stressors increases vulnerability to adverse health outcomes [5]. For the caregivers, stress can arise from the associated financial responsibilities, role changes, assisting in activities of daily living (ADL) and dealing with potential behavourial problems in caring for the frail care recipients [6]. Caregiver stress is a consequence of several interrelated events and directly related to hardship and problems experienced in caregiving and role strain outside of caregiving [7]. Long term demands for caregiving can result in decline in psychological health, anxiety [8], maladaptive coping [9], decreased care provision and decreased quality of life for both caregivers and care recipients [10].

Competency refers to the skills needed by an individual to perform a task at a certain level of performance [11]. A study showed that high competence had protective effect against psychological burden of caregiving for caregivers of persons with dementia and suggested there may be similar positive association for caregivers of frail older adults [8]. However, previous care experience was not associated with improved caregiving effectiveness if the complexity of care needs of a dependent person rises. This in turn, results in a higher level of burden and sense of insecurity [12].

Positive aspects of caregiving (PAC) has mediating effects on caregiver burden [13]. PAC optimizes positive experience and reduces stress as it promotes a sense of personal accomplishment and gratification, increases family cohesion, sense of personal growth and purpose in life [14].

Social support is a multi-dimensional construct which comprises instrumental support, such as help with daily tasks; informational support and emotional support: such as having someone to discuss problems [15]. Social support may refer to actual received specific support, or perceived, that is, the subjective appraisal of adequacy and quality of support. A meta-analysis found that perceived social support has a greater effect on caregiver burden than received support [16].

Variations of the definition of multimorbidity existed in different literature with most literature citing as the coexistence of two or more chronic conditions [17]. Our study adopted the cut- off of multimorbidity as three or more chronic medical conditions based on a systematic review [18]. Using three or more chronic medical conditions enabled more discrimination in identifying patients with higher needs, and was found to be more clinically useful and specific to older adults [19]. In Singapore, local prevalence of multimorbidity was found to be higher in patients with increasing age, male gender, and those of Chinese and Indian ethnicity [20]. As compared to a non- Chinese primary caregiver, a Chinese primary caregiver had a 3 times odds of perceiving burden [1].

Older adults with both frailty and multimorbidity may increase in caregiver burden but presence of perceived caregiving competence, positive aspects in caregiving and social support may alleviate this burden. There has been limited research on caregiver burden of caregivers of frail, multi-morbid older adults, particularly in Asian context. The impact of psychosocial factors on caregiver burden for this population has not been explored. Hence, we conducted this study based on the original research from Ding et al. (2022) [1] study, and further explored the psychosocial aspects of family caregiver burden using the existing data.

### Aim

The aims of the study were.

- To determine the difference in perceived caregiving competence, perceived positive aspects of caregiving and perceived social support between primary family caregivers of frail older adults with multimorbidity who perceived caregiver burden versus those who perceived no caregiver burden
- To determine the psychosocial factors associated with caregiver burden of primary family caregivers of frail older adults with multimorbidity

### Research hypothesis

We hypothesized that caregivers who perceived caregiver burden would have lower perceived caregiving competence, lower perceived positive aspects of caregiving and lower perceived social support.

## Methods

- Study Design

This was a descriptive cross-sectional study utilizing interviewer-administered questionnaires.

- Setting and Participants

The ‘rule of thumb’ states that a minimum of 10 participants per predictor variable is needed to determine sample size for regression equations [21]. Hence, for 18 independent variables, a minimum of 180 participants was needed. Participants were recruited from two public primary care clinics (Polyclinics) in Singapore over 5 months from July 2020 to December 2020. Polyclinics served to provide a one-stop health service centre providing outpatient medical treatment, health promotion and chronic disease management. Consecutive recruitment was done for caregivers who fulfilled the criteria of informal caregiving for at least three months, (i.e., they did not receive a salary for caregiving), were family members of the care recipients, were primary caregivers most involved in providing or ensuring care for care recipients and were able to speak English or other local languages. Their care recipients were aged 65 and above, not institutionalized, had multimorbidity with at least three chronic medical conditions from Fortin et al., 2017 [22] list of conditions for primary care, with a Clinical Frailty Scale (CFS) [23] score 4 and above, indicating mild to severe frailty. Those who were receiving cancer treatment or palliative care were excluded as their specific caregiving needs were not within the purview of primary care.

- Ethical Considerations

The study was approved by the National Healthcare Group- Domain Specific Review Board (NHG DSRB Ref: 2020/00014). Verbal informed consent was obtained from all participants.

- Data Collection

Questionnaires were administered by trained study team members proficient in the participants’ spoken language in the primary care clinics. Data collected were entered by the study team electronically via the Research Electronic Data Capture (REDCap ®), an intranet accessible platform [1]. REDCap ® is a web- based software developed by Vanderbilt University that capture research data in a systematic manner [24].

- Measures

The questionnaire comprised of caregivers’ sociodemographic variables such as age, gender, education, working status, presence of alternative caregivers and use of community resources. The perceived caregiving competence, positive aspects of caregiving and social support were measured using a validated questionnaire- Caregiving Competence Scale (CCS) [8], Short Positive Aspects of Caregiving (S-PAC) [25] and the modified Medical Outcomes Study Social support survey (mMOS-SS) [23] respectively. Caregiver burden was measured using the 12-item Zarit Burden Interview (ZBI) [27].

The CCS is a four-item scale that measures self-appraisal of one’s efficacy in caregiving. Scoring ranged from not at all (1) to very much (4). A possible score ranged from 4 to 16 with higher scores corresponding to higher levels of competency in caregiving. The CCS has Cronbach’s alpha of 0.74 suggesting it has good internal consistency. It was used locally for caregivers of frail persons in a hospital context [8]. A Chinese version of CCS has been developed and psychometric properties evaluated on family caregivers of stroke survivors. It also demonstrated good internal consistency (Cronbach’s alpha of 0.81) [28].

The S- PAC consists of seven items, which was validated among a more general caregiver population comprising caregivers of home dwelling older adults with functional limitations [25] and frailty [29]. Five items of the self-affirmation (SA) subscale and two items on the outlook-on-life (OL) subscale are scored on a five-point Likert scale ranging from disagree a lot (1) to agree a lot (5). A higher score indicates a more positive perception of caregiving experience. The S-PAC demonstrated good internal consistency in our local setting with a Cronbach’s alpha of 0.91 (overall scale) [25].

The 8- item mMOS-SS consists of two subscales covering two domains (emotional and instrumental [tangible] social support) with four items each. Participants were asked on how often social support such as companionship or assistance were available to them when they needed it. It was measured on a 5- points Likert scale ranging from none of the time (1) to all of the time (5). Scores were calculated as the average score of subscale items transformed to a zero to 100 scale with higher scores indicating more support. The mMOS-SS was a valid and reliable measure of social support especially for geriatric assessments [26]. Across populations, the internal consistency of the mMOS-SS measure was very good (Cronbach’s alpha 0.88 to 0.93).

The 12-item ZBI measured subjective caregiver burden. It scores ranged from 0 to 48, with higher score indicating greater burden. Each item was scored on a 5- point Likert scale from 0 to 4 (never to nearly always). For this study, a cut off score of 10 or above was used to determine that family caregivers perceived caregiver burden. This cut-off was based on the study of caregivers of frail older persons by Mello and team [30] as this was most similar to our population of interest. The 12-item ZBI was validated in other caregiver populations such as informal caregivers of elderly persons irrespective of level of cognition (Cronbach’s alpha 0.90) [27], caregivers of community-dwelling older adults with diverse comorbidities (Cronbach’s alpha 0.81) [31] and caregivers of frail older persons [30]. The 12-item ZBI was also used and validated locally, but for caregivers of persons with dementia [32].

A pilot study of 20 participants was conducted to determine face validity and test–retest reliability of the overall questionnaire in local context. The questionnaire was understandable and no amendments were needed. The intraclass correlation (ICC) for test–retest reliability was 0.932 (CI 0.919, 0.937, p < 0.001) indicating excellent reliability [33].

### Data analysis

Data was extracted from the REDCap® database. Demographic information was reported using descriptive statistics. Normality was not met and thus Mann–Whitney U test was used to compare differences in caregiver competence, PAC and social support scores between caregivers who perceived burden and those who did not. Multivariable analysis was used to analyze the impact of all psychosocial factors on caregiver burden. Statistical significance was set at a p-value less than 0.05. Statistical Package for the Social Sciences (SPSS) version 26 was used for all the analysis.

## Results

A total of 205 eligible caregivers were approached, of whom 188 agreed to participate, giving a response rate of 91.7%. The characteristics of the caregivers and total mean score for CCS, S-PAC, mMOS-SS and ZBI score are shown in Table 1.

**Table 1:** Characteristics of Caregivers

Characteristic	^a^N = 188 (%)
Age, years	
Median (Interquartile range- IQR)	62.0 (52.0—70.0)
Gender	
Male	66 (35.1)
Female	122 (64.9)
Ethnicity	
Chinese	158 (84.0)
^ b^Non-Chinese	30 (16.0)
Marital Status	
Never Married	45 (23.9)
Married	132 (70.2)
Separated/ Divorced/ Widowed	11 (5.9)
Highest Level of Education completed	
No formal education	20 (10.6)
Primary education	42 (22.3)
Secondary education	70 (37.2)
Tertiary education	56 (29.8)
Main Work Status, over the last 12 months	
Full-time work	73 (38.8)
Part-time work	29 (15.4)
Homemaker	49 (26.1)
Retired/ Unemployed/ Student	37 (19.7)
Relationship with Care Recipient	
Spouse	50 (26.6)
Child	112 (59.6)
Others	26 (13.8)
Dwelling	
HDB 1 to 3 room flat	80 (42.6)
HDB 4 or 5 room or executive flat	94 (50.0)
Condominium/ landed property/ others	14 (7.4)
Living with Care Recipient	
Yes	126 (67.0)
No	62 (33.0)
Receiving Financial Support	
Yes	57 (30.3)
No	131 (69.7)
Duration of Caregiving, years	
Median (IQR)	5.0 (3.0–10.0)
Time Spent Caregiving per week, hours	
Median (IQR)	20.0 (12.0—30.0)
Use of Community Resources in past 3 months	
Centre based services	24 (12.8)
Home care services	9 (4.8)
Caregiver training/ support	5 (2.6)
None	150 (79.8)
Presence of Alternative Caregivers	
Family only	48 (25.6)
Foreign Domestic Worker (FDW) only	51 (27.1)
Both family and FDW	16 (8.5)
None	73 (38.8)
Perceived Caregiving Competency Scale (CCS) Score	
Median (IQR)	11 (8.0–12.0)
Perceived Positive Aspects of Caregiving (S-PAC) Score	
Median (IQR)	28 (25.0–31.0)
Perceived Social Support (mMOS-SS) Score	
Median (IQR)	28 (22.0–32.0)
Zarit Burden Score	
Median (IQR)	15 (9.0–22.0)
No burden perceived (ZBI score < 10)	53 (28.2)
Burden perceived (ZBI score ≥ 10)	135 (71.8)

^a^Total number = 188, ^b^Non-Chinese included Malay, Indian and other ethnicities

Table 2 presents the individual item scores for perceived caregiving competence, positive aspects in caregiving and social support of the caregivers for all participants. Most of the respondents agreed that caregiving “made them feel useful”, “important” and “appreciated”. In terms of social support, more than one-third of caregivers had someone to turn to for suggestions most of the time.

**Table 2:** Individual item scores for CCS, S- PAC and mMOS-SS

Individual Item Score	^a^*N* = 188 (%)				
CAREGIVING COMPETENCE SCALE (CCS)	Not at all	Just a little	Somewhat	Very much	
1. How much do you believe that you've learned how to deal with very difficult situations?	11 (5.9)	48 (25.5)	101 (53.7)	28 (14.9)	
2. How much do you feel that all in all, you're a good caregiver?	18 (9.6)	50 (26.6)	96 (51.1)	24 (12.8)	
3. How competent do you feel?	18 (9.6)	60 (31.9)	95 (50.5)	15 (7.98)	
4. How self-confident do you feel?	26 (13.8)	65 (34.6)	87 (46.3)	10 (5.32)	
SHORT POSITIVE ASPECTS OF CAREGIVING SCALE (S- PAC)					
Providing help/care to or ensuring provision of care has…	Disagree a lot	Disagree a little	Neither agree nor disagree	Agree a little	Agree a lot
1. made me feel more useful	0 (0)	7 (3.7)	13 (6.9)	108 (57.5)	60 (31.9)
2. made me feel needed	0 (0)	5 (2.7)	10 (5.3)	89 (47.3)	84 (44.7)
3. made me feel appreciated	3 (1.6)	10 (5.3)	33 (17.6)	89 (47.3)	53 (28.2)
4. made me feel important	2 (1.1)	3 (1.6)	19 (10.1)	109 (57.9)	55 (29.3)
5. made me feel strong and confident	6 (3.2)	19 (10.1)	63 (33.5)	81 (43.1)	19 (10.1)
6. enabled me to appreciate life more	2 (1.1)	13 (6.9)	29 (15.4)	81 (43.1)	63 (33.5)
7. strengthened my relationships with others	8 (4.3)	22 (11.7)	56 (29.8)	63 (33.4)	39 (20.7)
SOCIAL SUPPORT SURVEY INSTRUMENT (mMOS- SS)	None of the time	A little of the time	Some of the time	Most of the time	All of the time
1. Someone to turn to for suggestions about how to deal with a personal problem	10 (5.3)	27 (14.4)	42 (22.3)	85 (45.2)	24 (12.8)
2. Someone who understands your problems	11 (5.9)	30 (15.9)	53 (28.2)	73 (38.9)	21 (11.2)
3. Someone to help you if you were confined to bed	26 (13.8)	25 (13.3)	40 (21.3)	74 (39.4)	23 (12.2)
4. Someone to take you to the doctor if you needed it	22 (11.7)	23 (12.2)	42 (22.3)	72 (38.3)	29 (15.4)
5. Someone to prepare your meals if you were unable to do it yourself	26 (13.8)	20 (10.6)	42 (22.3)	74 (39.4)	26 (13.8)
6. Someone to help with daily chores if you were sick	24 (12.8)	20 (10.6)	40 (21.3)	76 (40.4)	28 (14.9)
7. Someone to love and make you feel wanted	15 (7.9)	31 (16.5)	41 (21.8)	74 (39.4)	27 (14.4)
8. Someone to have a good time with	13 (6.9)	27 (14.4)	52 (27.7)	70 (37.2)	25 (13.3)

Mann–Whitney U test was used to compare differences in perceived caregiving competency, positive aspects of caregiving and social support between caregivers who perceived burden versus those who did not perceive burden. As shown in Table 3, caregivers who perceived burden had significantly lower perceived caregiving competency, significantly lower perceived positive aspects of caregiving and significantly lower perceived social support.

**Table 3:** Comparison of CCS, S-PAC and mMOS-SS between caregivers who perceived versus did not perceive burden

	Burden not perceived Median (IQR)	Burden perceived Median (IQR)	*p*- value
Perceived Caregiving Competency Scale (CCS)	12.0 (9.5- 14.0)	10.0 (8.0- 12.0)	< 0.001*
Perceived Positive Aspects of Caregiving (S-PAC)	30.0 (27.5- 34.0)	27.0 (24.0- 30.0)	< 0.001*
Perceived Social Support (mMOS-SS)	32.0 (28.5- 34.0)	26.5 (16.8- 32.0)	< 0.001*

Multivariable logistic regression was used to analyse factors associated with caregiver burden (Table 4). Factors significantly associated with higher odds of perceived burden were living in 4 or 5 room housing development board (HDB) or executive flats (OR 4.4, 95% CI 1.39, 13.72, *p* = 0.01), presence of alternative caregivers (OR 3.3, 95% CI 1.09, 10.19, *p* = 0.04), use of community resources (OR 4.4, 95% CI 1.15, 16.83, *p* = 0.03) and time spent caregiving per week (OR 1.1, 95% CI 1.02, 1.10, *p* = 0.003). Higher caregiving competency (OR 0.7, 95% CI 0.58, 0.96, *p* = 0.02) and social support (OR 0.8, 95% CI 0.74, 0.89, *p* < 0.001) were significantly associated with lower odds of perceived burden. No statistically significant differences were found between caregiver ZBI score and age, gender, marital status, educational level and employment status of caregiver. Also, no difference was found in terms of relationship with (child vs spouse), or living with care recipient, or duration of caregiving with ZBI score.

**Table 4:** Factors associated with caregiver burden as measured by ZBI score

	Odds Ratio	95% CI^#^. for EXP(B)		^∞^Sig
		Lower	Upper	
Age of caregiver	1	0.95	1.06	0.99
Gender				
Male	REF^ǂ^			
Female	0.5	0.17	1.46	0.20
Ethnicity				
Chinese	REF			
Non-Chinese	0.6	0.18	2.22	0.47
Marital Status				
Never Married	REF			
Married	2.4	0.53	10.91	0.25
Separated/ Divorced/ Widowed	0.6	0.07	5.61	0.66
Education				
No Formal Education	REF			
Primary	5.2	0.79	34.31	0.09
Secondary	5.0	0.63	39.37	0.13
Diploma/University	4.3	0.46	39.33	0.20
Main work status over the last 12 months				
Full-time work	REF			
Part-time work	0.6	0.13	3.16	0.59
Homemaker	1.1	0.22	5.80	0.88
Unemployed/ Student/ Retired	1.3	0.24	7.48	0.75
Relationship with the care recipient				
Spouse	REF			
Child	2.2	0.35	13.71	0.40
Others	4.5	0.43	48.26	0.21
Dwelling				
HDB 1–3 room flat	REF			
HDB 4/5 room/ Executive flat	4.4	1.39	13.72	0.01*
Condominiums/ Landed property/ others	0.3	0.04	2.21	0.24
Living with Care Recipient				
No	REF			
Yes	0.8	0.27	2.42	0.69
Receiving Financial Support				
No	REF			
Yes	1.4	0.32	6.43	0.63
Presence of Alternative Caregivers				
No	REF			
Yes	3.3	1.09	10.19	0.04*
Use of community resources in the last 3 months				
No	REF			
Yes	4.4	1.15	16.83	0.03*
Duration of Caregiving, years	0.9	0.87	1.04	0.31
Time Spent Caregiving per week, hrs	1.1	1.02	1.10	0.003*
Caregiving Competence Scale Score	0.7	0.58	0.96	0.02*
Positive Aspects of Caregiving Scale Score	1	0.87	1.17	0.91
Social Support Survey Score	0.8	0.74	0.89	< 0.001*

^#^*CI* Confidence interval, ^ǂ^*REF* Reference, ^∞^*Sig* Significance value

## Discussion

The aim of our study was to evaluate to what extent psychosocial factors can mitigate the effect of burden experienced by family caregivers of frail older adults with multimorbidity. In the present study of 188 caregivers, we found that caregivers who perceived burden had significantly lower perceived caregiving competence, positive aspects of caregiving and social support scores. In addition, presence of alternative caregivers, community resources and longer time spent in caregiving were associated with caregiver burden.

### Sociodemographic factors

Taking into account of our study population sociodemographic factors, where a large proportion were middle- aged females still in the workforce, the feeling of burden could arise from both work and caregiving duties for their frail elderly family members. Although most of the caregivers were adult child (vs spouse) to their care recipient, we found no association between their level of burden and the relationship to or living with the care recipients. The higher odds of burden for those living in 4 or 5 room HDB flats compared to those living in smaller 1 to 3 room HDB flats could be related to a larger household size which included foreign domestic workers (FDWs). The local survey on caregiving found that 5 room flats and larger had higher proportions of FDWs for care recipients. The need for FDWs could reflect higher care needs of care recipients and thus higher burden (34).

### Perceived caregiving competence

In terms of caregiving competence, our study demonstrated that perceived competence was associated with lower odds of perceived burden. This finding is comparable to a recent study done among 274 older adults family caregivers for frail older adults in Singapore, which showed caregiving competency could reduce negative psychological stressors and result in lower burden [8]. Another study in the United States (US) also found that among caregivers of older adults with multimorbidity, those who had higher perceived self-efficacy and lower perceived health care task difficulty had lower caregiver strain and depression [35]. A study in the US also found that caregiving mastery, or caregivers’ perceived ability in caregiving was a key factor in determining caregiver burden [36].

### Perceived positive aspects in caregiving

Although bivariate analysis showed caregivers who did not perceive burden had higher perceived PAC, this was not significant in the logistic regression. Demographic factors that influenced PAC included informal caregivers with lower educational level and caregivers of Malay ethnicity as these were significantly associated with higher PAC [37]. Being an immediate family caregiver (adult–child or spousal caregiver) versus distant family caregiver (siblings, nephews, niece) were associated with lower PAC [37]. Our study did not find such association with caregiver burden. Previous studies have found mixed results for PAC and burden. Wong and colleagues (2019) [29] found that while wife caregivers reported lower PAC, Having PAC significantly lowered the effects of caregiving burden on psychological distress. In contrast, this effect was not significant for husband caregivers. In Japan, researchers suggested that a sense that life was worth living had an important role in preventing the development of caregiver burden [38]. A qualitative study of family caregivers of older adults in Canada found that despite physical, mental, emotional, and/or financial challenges faced by caregiving participants, they were able to find meaning in their caregiving role [39].

### Social support

Our study showed that more time spent caregiving per week was associated with higher odds of burden. We found that caregivers spent an average of 20 h per week and this was similar to a local study in which informal caregiving time was 19.7 h [40]. One study demonstrated that obligation to caregiving likely resulted in less time allocation for oneself leisure activity, hobbies and social life, which could affect well- being and life satisfaction of the caregiver in general [41]. Similarly, a local study on informal caregivers reported that the impact of caregiving had on caregivers may include disruption to one’s own schedule, health and financial problems arising from caregiving [34]. We believed that having respite or “time off” from caregiving duties may improve social well- being of the caregiver and potentially alleviate caregiver burden.

This study found that perceived social support was associated with reduced odds of perceived burden. This finding is in line with a meta-analysis which found that perceived social support is consistently related to subjective burden [42]. Specifically, in Japan, emotional support from caregivers’ family members was found to be essential for better caregiver subjective health [43]. A recent study in Spain also found that perceived social support was associated with better mental well-being in older caregivers [44]. In Italy, perceived support from both family members and friends was associated with better health related quality of life for patients with multiple chronic conditions and their informal caregivers [45]. A study of caregivers of older adults in Singapore also found that perceived social support mediated the association between resilience and caregiver burden [46].

Interestingly, caregivers who had alternative caregivers (either another member of the family or FDW) were more likely to perceive burden in this study. This was an unanticipated finding as it was assumed that the availability of additional caregivers would provide respite and alleviate burden for the main caregivers. A possible explanation could be that the need to engage an alternative caregiver may have been due to care recipients’ increasing frailty. Schulz and team [4] suggested that care dependence needs could evolve from basic to complex personal care, from taking care of recipients’ medical appointments and communicating with healthcare providers, to medication and symptoms monitoring, hiring domestic helpers and then further on to assisting in ADL [4]. Many of these activities warrant additional time and resources with higher level of complexity, thus, also increasing stress for the caregiver [4]. The presence of alternative family caregivers may also have led to conflict. In a qualitative study in Canada, adult children caregivers revealed sibling conflicts and differences in opinions that could deteriorate family relationships, therefore resulting in greater perceived burden [39]. In addition, varying competence levels of alternative caregivers such as FDWs may have added to burden. A local study found that FDWs may not be equipped with specific knowledge and confidence to perform health related tasks when caring for older adults [34].

In this study, caregivers who utilized community resources within the past three months were more likely to perceive burden. As with the case of the need for alternative caregiver, a possible explanation was that increasing care dependence of the care recipient necessitated utilization of community resources, which in turn posed additional financial burden [47]. This was in contrast to other studies which found that formal community resources reduced burden [36, 48]. However, the percentage of caregivers who utilized community resources in this study was very low, which was similarly found in previous study in Singapore [34]. While there may be expanded capacity and options of care services available, awareness of available services could be lacking and navigation through these support services could be challenging for some. In a study in Japan of 46 pairs of caregiver-elderly dyads, caregiver who reported inconvenience to use care services reported higher burden than those who did not [49].

## Conclusion

Our study illustrated that psychosocial factors (perceived caregiving competence and social support) influenced caregiver burden among older adults with frailty and multimorbidity. These findings enhanced our understanding of caregiver burden with increasing care dependence (frailty) of our elderly population in primary care setting. This highlights the need for clinicians to assess and address psychosocial factors associated with caregiver burden. These include competence needs of both primary and alternative caregivers as well as caregivers’ awareness of community resources and formal support services. Policy makers should incorporate caregivers’ opinions to enhance resources for caregivers which are tailored to their needs. The findings added to evidence about the significance of perceived social support as a predictor of caregiver burden. The novel findings that alternative caregivers and the use of community resources may increase the likelihood of caregiver burden necessitate further research in this area. Whilst this study did not confirm the significant positive aspect of caregiving association with caregiver burden, more than half the caregivers reported positive feelings and felt being useful, appreciated and important when providing care. Future research could consider longitudinal study to evaluate the effects of psychosocial factors over time. Research could also evaluate interventions to improve PAC, caregiving competence and social support. It will also be useful to determine which aspects of caregiving are perceived to cause most burden, as addressing the root cause may further alleviate burden.

### Strengths and limitations

Our study has several strengths. It is of relevance locally and in the Asian context, as there are not many studies that explored psychosocial factors and caregiver burden in Asia. Reporting bias was minimized as this interviewer – administered study was carried out by a small team of three interviewers who had standardized the interview methods prior to the start of the research project. Also, ZBI used was general and not disease specific, hence it had a wider scope of capture for caregiver burden.

Our findings should be interpreted within the study context and design. Firstly, being a cross-sectional study, temporal associations between the independent and outcome variables cannot be made. Secondly, the use of convenience sampling limited the generalizability of the findings. However, we minimized the potential bias by inviting all eligible caregiver-care recipient dyads who attended the clinic during the recruitment period.

## Data Availability

The dataset and personal health data generated and/or analysed during the current study are not publicly available due to the Data Protection Act Commission Singapore—Advisory Guidelines for the Healthcare Sector and legal and ethical restrictions related to data privacy protection but are available from the corresponding author on reasonable request.
